# Supplementary material for: Flavonoids from Piper Species as Promising Antiprotozoal Agents against Giardia intestinalis: Structure-Activity Relationship and Drug-Likeness Studies
Source: Pharmaceuticals (Basel). 2022 Nov 10;15(11):1386. doi: 10.3390/ph15111386 (PMC9695682; doi:10.3390/ph15111386)
Supplement: Supplementary file 1 [file pharmaceuticals-15-01386-s001.zip › pharmaceuticals-2005852-supplementary.pdf]

Electronic Supporting Information

Flavonoids from *Piper* species as promising antiprotozoal agents against *Giardia intestinalis*. Structure-activity relationship and drug-likeness studies

Juan C. Ticona[a,b], Pablo Bilbao-Ramos[c], Ángel Amesty[a], Ninoska Flores[b], M. Auxiliadora Dea-Ayuela[c,d], Isabel L. Bazzocchi[a], Ignacio A. Jiménez[a,*]

[a] Instituto Universitario de Bio-Orgánica Antonio González and Departamento de Química Orgánica, Universidad de La Laguna, Avenida Astrofísico Francisco Sánchez 2, 38206 La Laguna, Tenerife, Spain.
[b] Instituto de Investigaciones Fármaco Bioquímicas, Facultad de Ciencias Farmacéuticas y Bioquímicas, Universidad Mayor de San Andrés, Avenida. Saavedra 2224, Miraflores, La Paz, Bolivia.
[c] Departamento de Parasitología, Facultad de Farmacia, Universidad Complutense de Madrid, Plaza Ramón y Cajal s/n, 28040-Madrid, Spain.
[d] Departamento de Farmacia, Bioquímica y Biología Molecular, Universidad CEU-Cardenal Herrera, Avda. Seminario s/n, 46113-Moncada, Valencia, Spain.

Table of contents

Page S3: **Figures S1 and S2**, ^{1}H NMR and ^{13}C NMR spectra of compound **1**.

Page S4: **Figures S3 and S4**, HSQC and HMBC spectra of compound **1**.

Page S5: **Figures S5, S6 and S7**, Mass spectrum of compound **1**, ^{1}H and ^{13}C NMR spectra of compound **2**.

Page S6: **Figures S8 and S9**, HSQC and HMBC spectra of compound **2**.

Page S7: **Figures S10, S11 and S12**, Mass spectrum of compound **2**, ^{1}H and ^{13}C NMR spectra of compound **3**.

Page S8: **Figures S13 and S14**, HSQC and HMBC spectra of compound **3**.

Page S9: **Figures S15, S16 and S17**, Mass spectrum of compound **3**, ^{1}H and ^{13}C NMR spectra of compound **4**.

Page S10: **Figures S18 and S19**, HSQC and HMBC spectra of compound **4**.

Page S11: **Figures S20, S21 and S22**, Mass spectrum of compound **4**, ^{1}H and ^{13}C NMR spectra of compound **5**.

Page S12: **Figures S23 and S24**, HMBC spectrum of compound **5** and Mass spectrum of compound **5**.

Page S13: **Figures S25, S26 and S27**, ^{1}H NMR ($CDCl_3$), ^{1}H NMR (C_6D_6) and ^{13}C NMR spectra of compound **6**.

Page S14: **Figures S28 and S29**, HSQC and HMBC spectra of compound **6**.

Page S15: **Figure S30**, Mass spectrum of compound **4**.

Page S16: **Table S1**, *In silico* ADME profile prediction of isolated flavonoids **1-29**.

Page S17-S22: **Experimental part S1**, phytochemical studies.

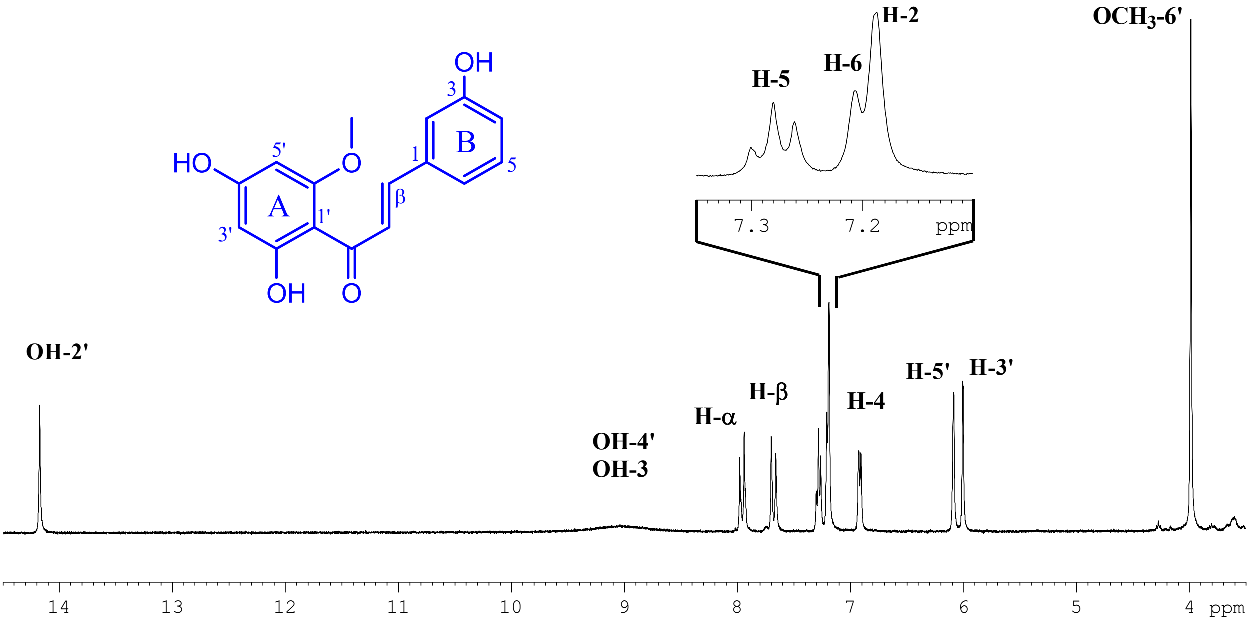

Figure S1. ^{1}H NMR spectrum [400 MHz, solvent $(CD_3)_2CO$] of compound **1**.

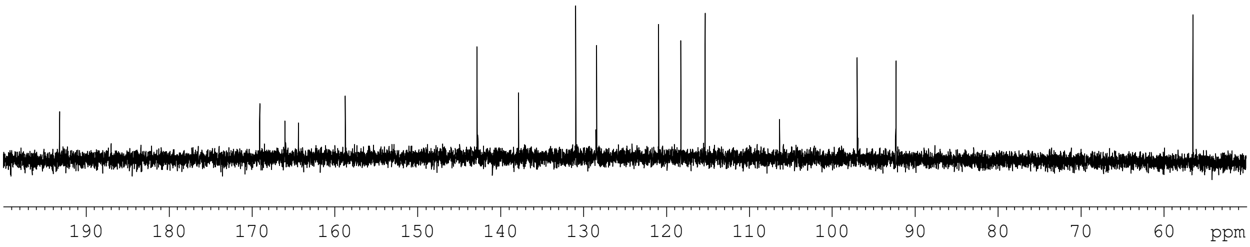

Figure S2. ^{13}C NMR spectrum [100 MHz, solvent $(CD_3)_2CO$] of compound **1**.

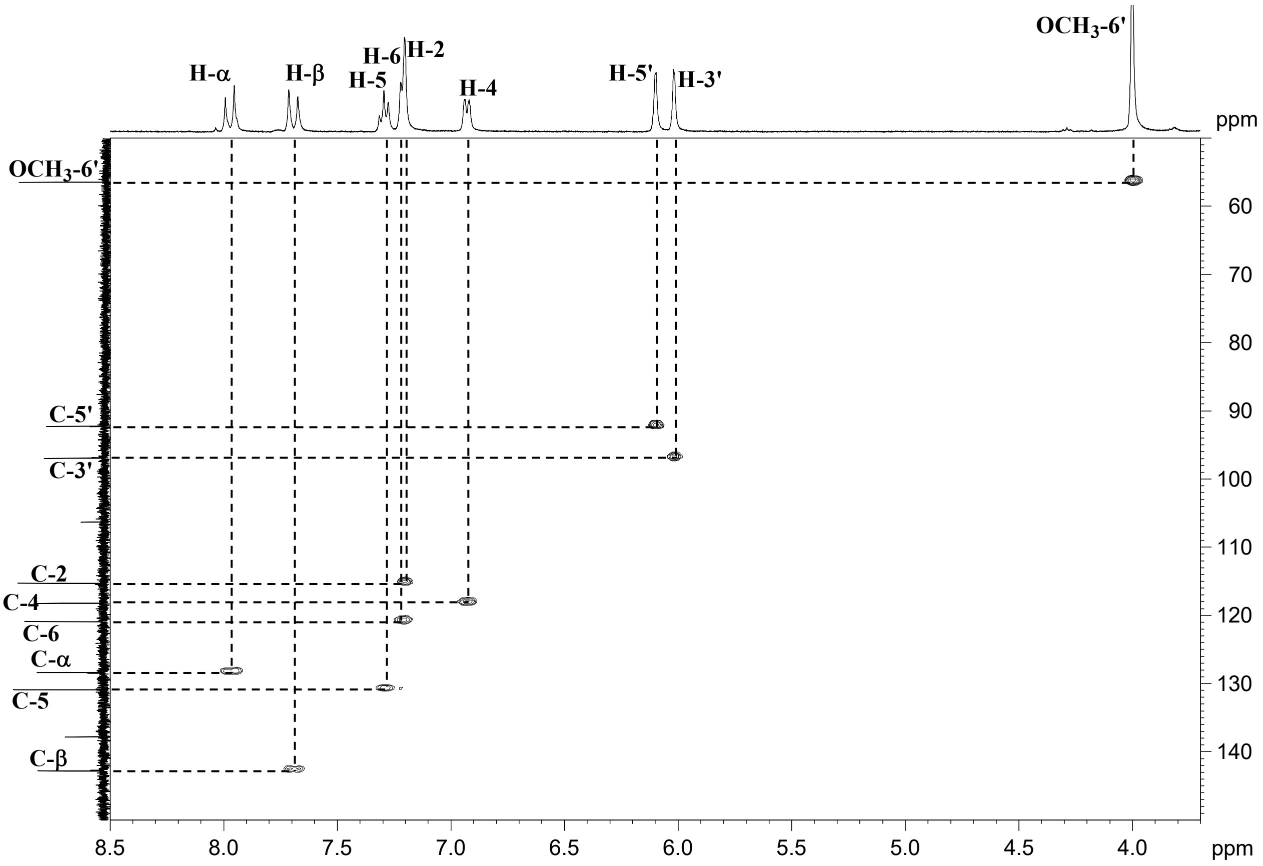

Figure S3. HSQC spectrum [400 MHz, solvent $(CD_3)_2CO$] of compound **1**.

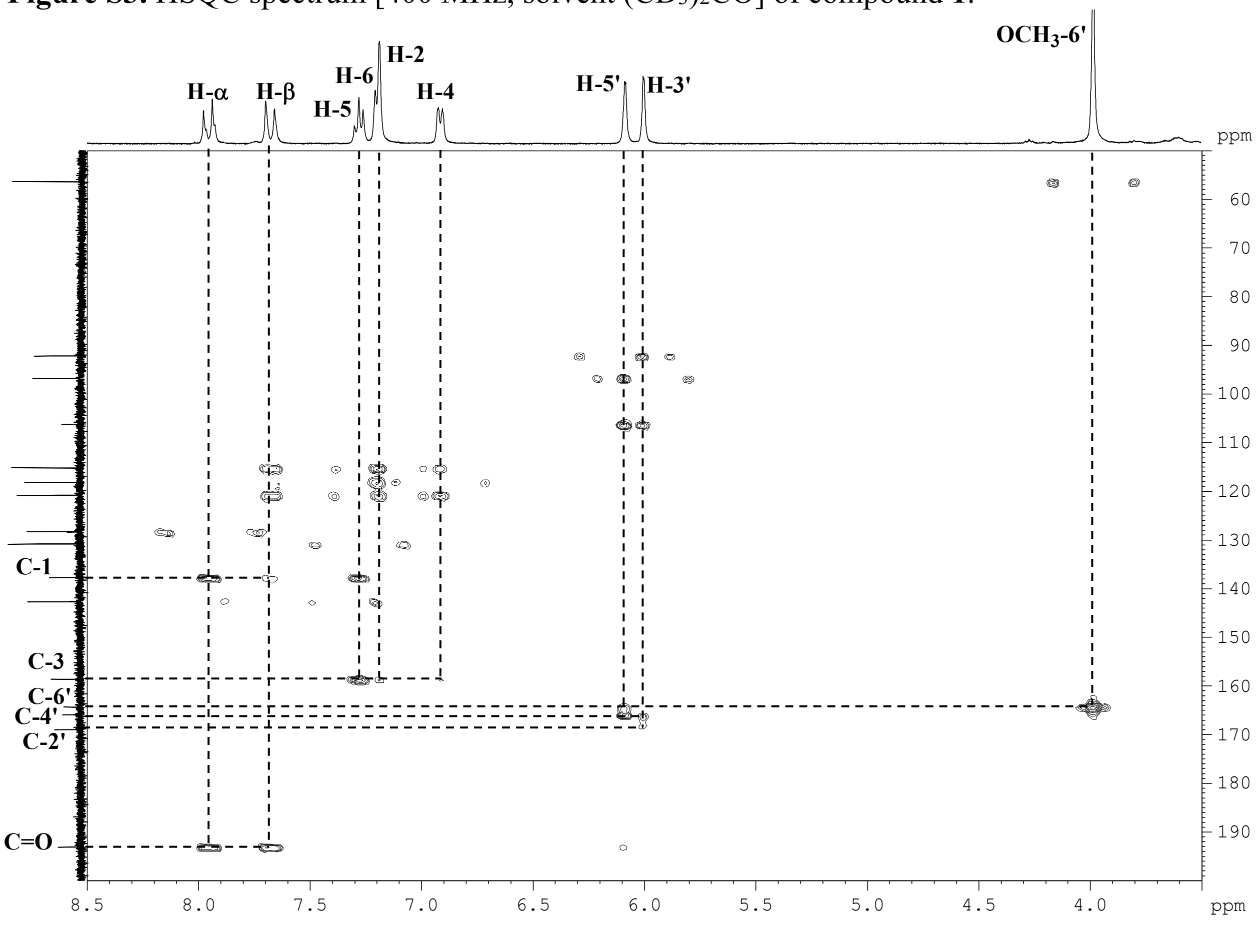

Figure S4. HMBC spectrum [400 MHz, solvent $(CD_3)_2CO$] of compound **1**.

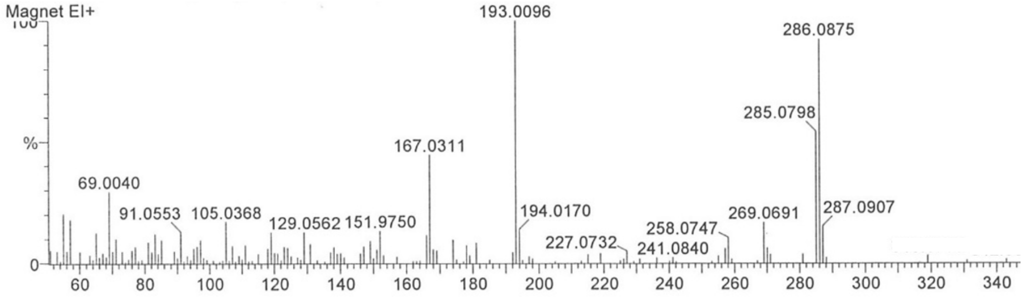

	Mass	RA	Calc. Mass	mDa	PPM	DBE	i-FIT	Formula
Minimum:		0.10				-1.5		
Maximum:		100.00		5.0	19.0	50.0		
	287.0907	15.08	287.0919	-1.2	-4.2	9.5	2773047.3	C16 H15 O5
	286.0875	92.37	286.0841	3.4	11.9	10.0	3.3	C16 H14 O5
	285.0798	54.17	285.0763	3.5	12.3	10.5	1139.3	C16 H13 O5

Figure S5. Mass spectrum of compound **1**.

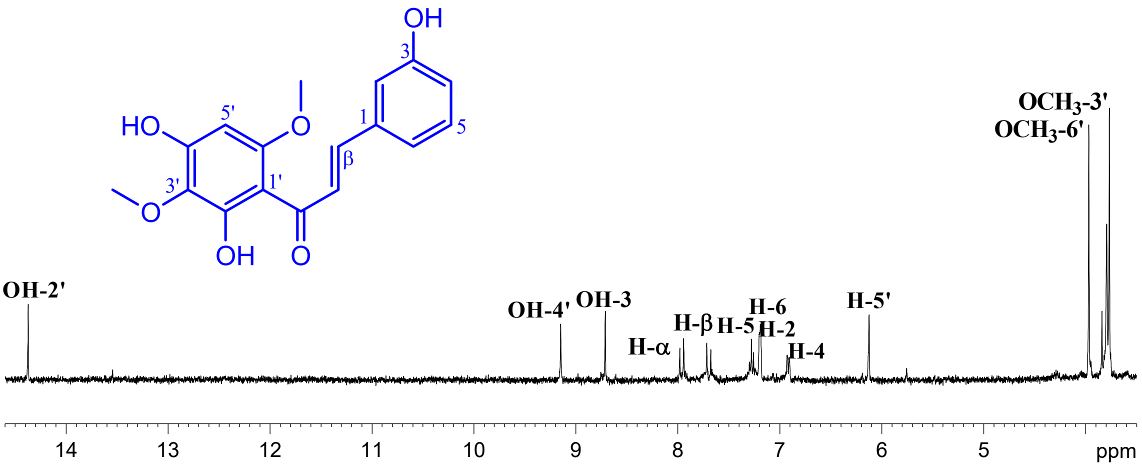

Figure S6. ^{1}H NMR spectrum [400 MHz, solvent $(CD_3)_2CO$] of compound **2**.

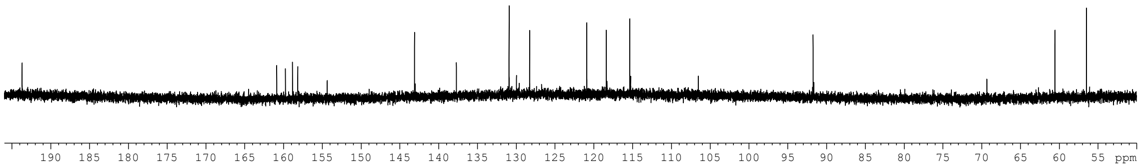

Figure S7. ^{13}C NMR spectrum [100 MHz, solvent $(CD_3)_2CO$] of compound **2**.

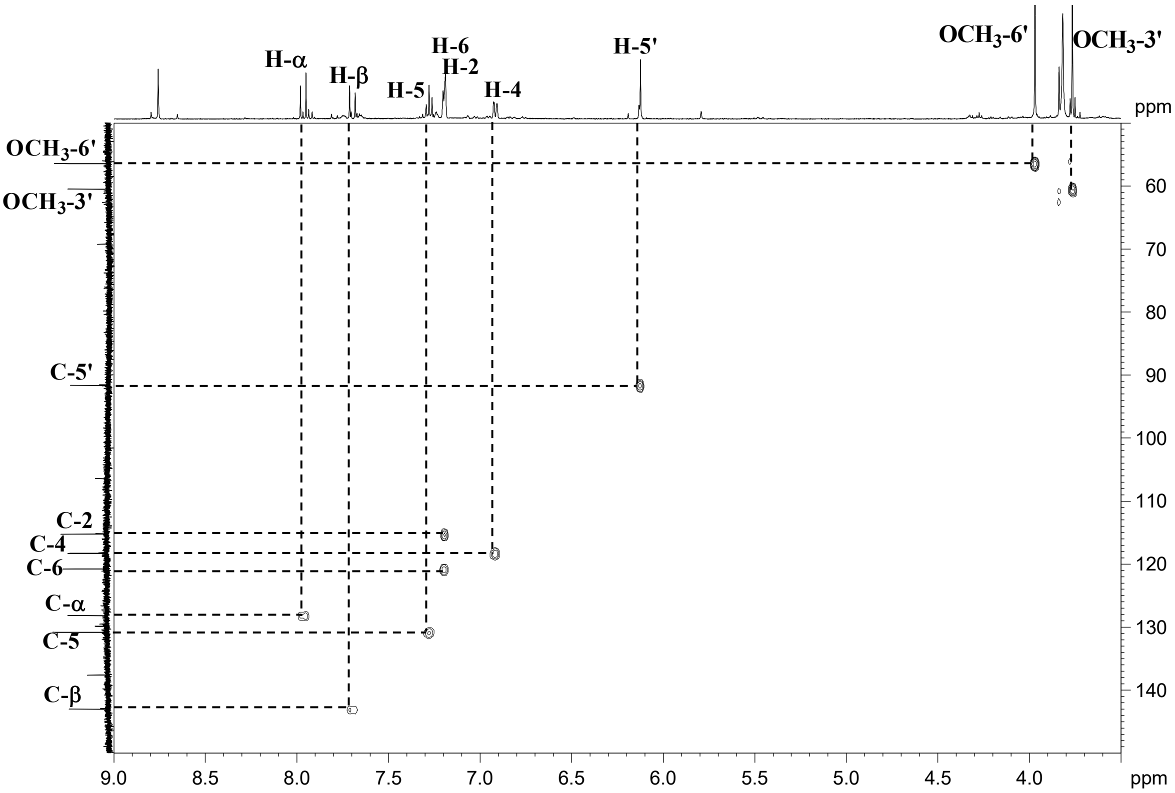

Figure S8. HSQC spectrum [400 MHz, solvent $(CD_3)_2CO$] of compound **2**.

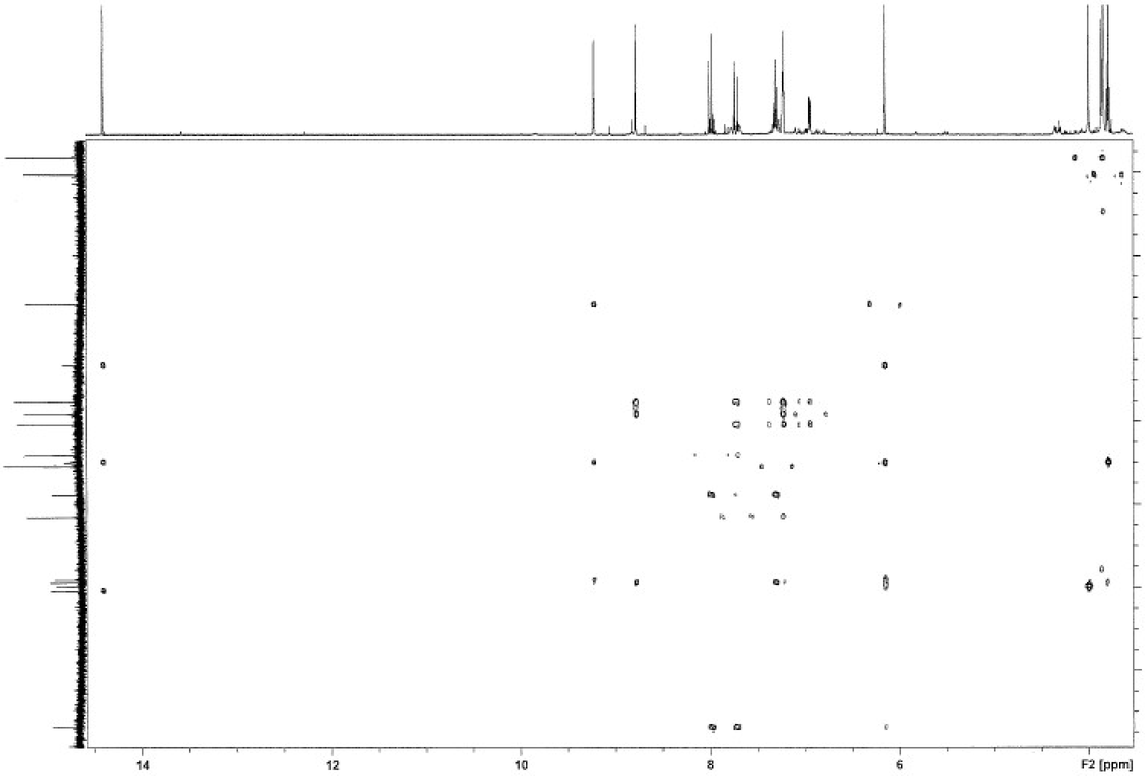

Figure S9. HMBC spectrum [400 MHz, solvent $(CD_3)_2CO$] of compound **2**.

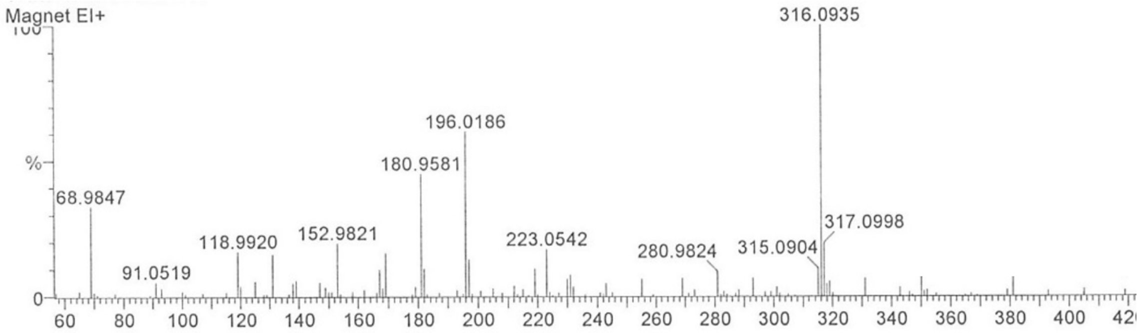

Minimum:	0.10				-1.5		
Maximum:	100.00		5.0	15.0	50.0		
Mass	RA	Calc. Mass	mDa	PPM	DBE	i-FIT	Formula
316.0935	100.00	316.0947	-1.2	-3.8	10.0	6.7	C17 H16 O6

Figure S10. Mass spectrum of compound **2**.

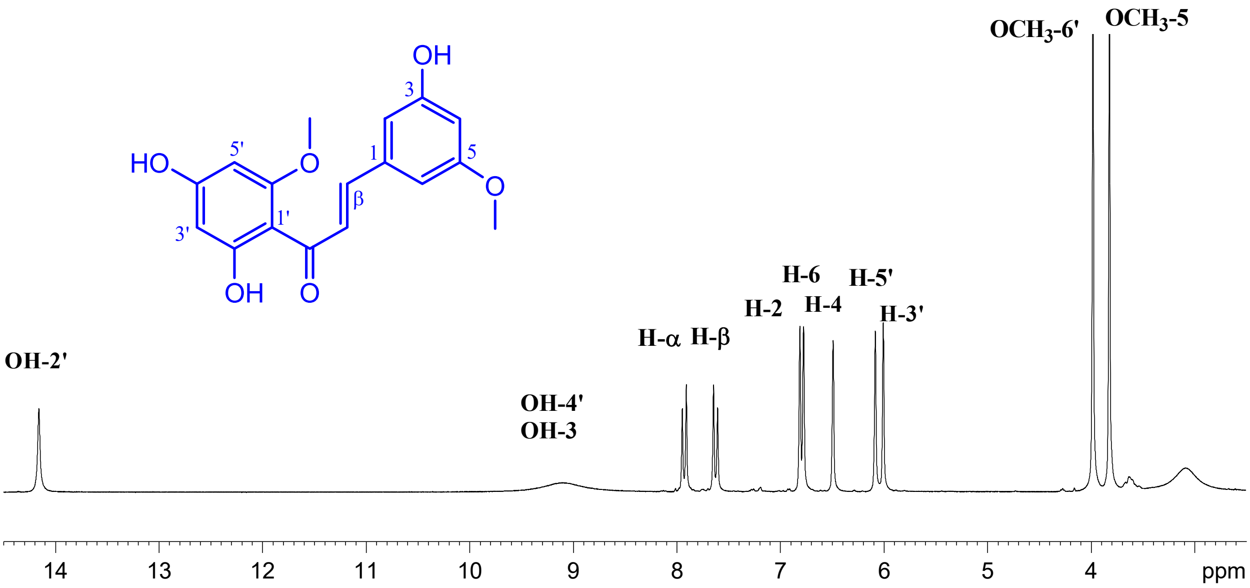

Figure S11. ^{1}H NMR spectrum [400 MHz, solvent $(CD_3)_2CO$] of compound **3**.

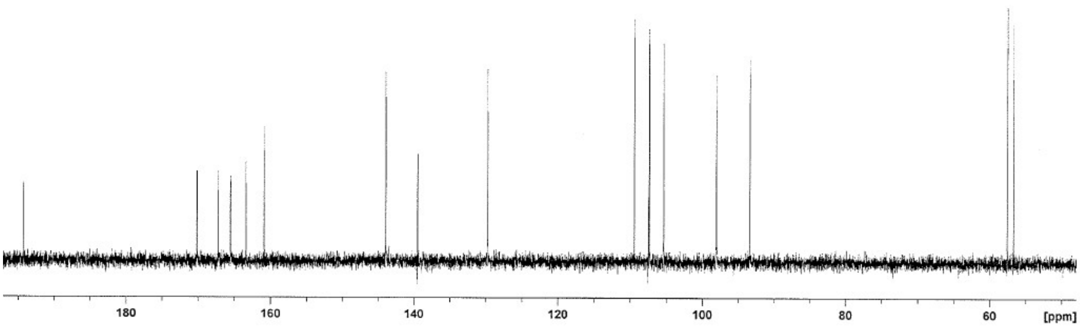

Figure S12. ^{13}C NMR spectrum [100 MHz, solvent $(CD_3)_2CO$] of compound **3**.

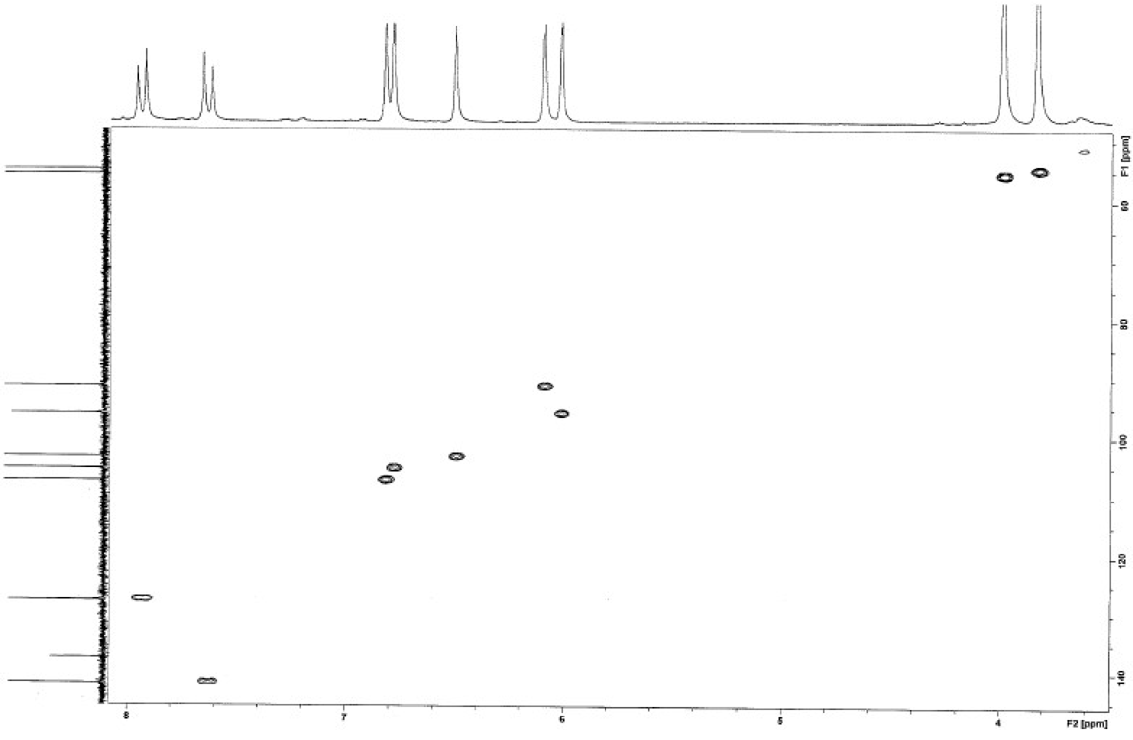

Figure S13. HSQC spectrum [400 MHz, solvent $(CD_3)_2CO$] of compound **3**.

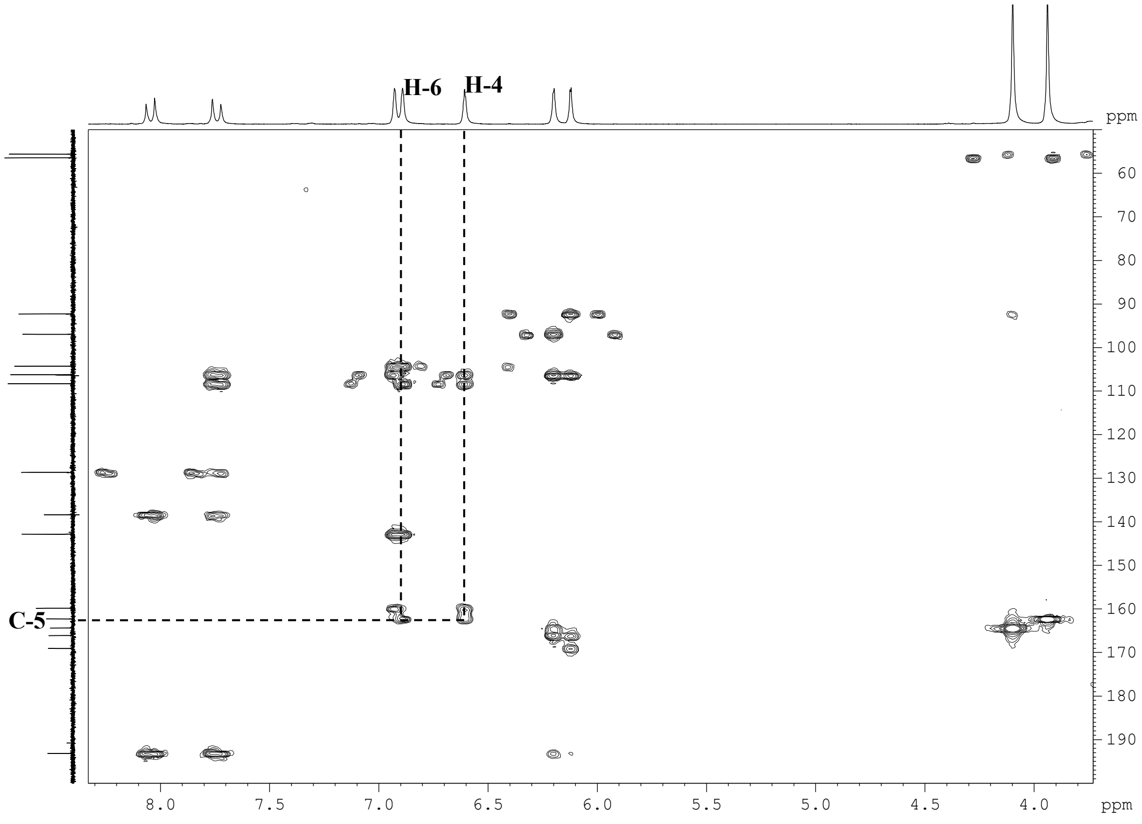

Figure S14. HMBC spectrum [400 MHz, solvent $(CD_3)_2CO$] of compound **3**.

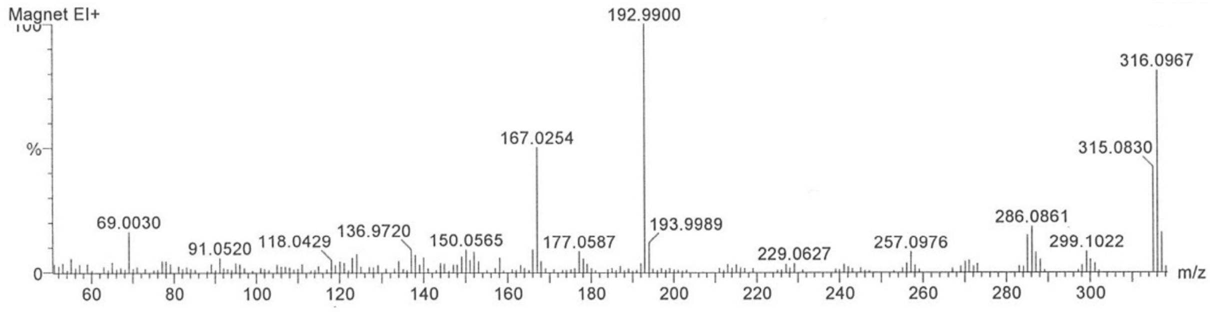

Minimum:	5.00				-1.5		
Maximum:	100.00		5.0	19.0	50.0		
Mass	RA	Calc. Mass	mDa	PPM	DBE	i-FIT	Formula
317.1009	16.09	317.1025	-1.6	-5.0	9.5	2774638.8	C17 H17 O6
316.0967	81.31	316.0947	2.0	6.3	10.0	6.6	C17 H16 O6

Figure S15. Mass spectrum of compound **3**.

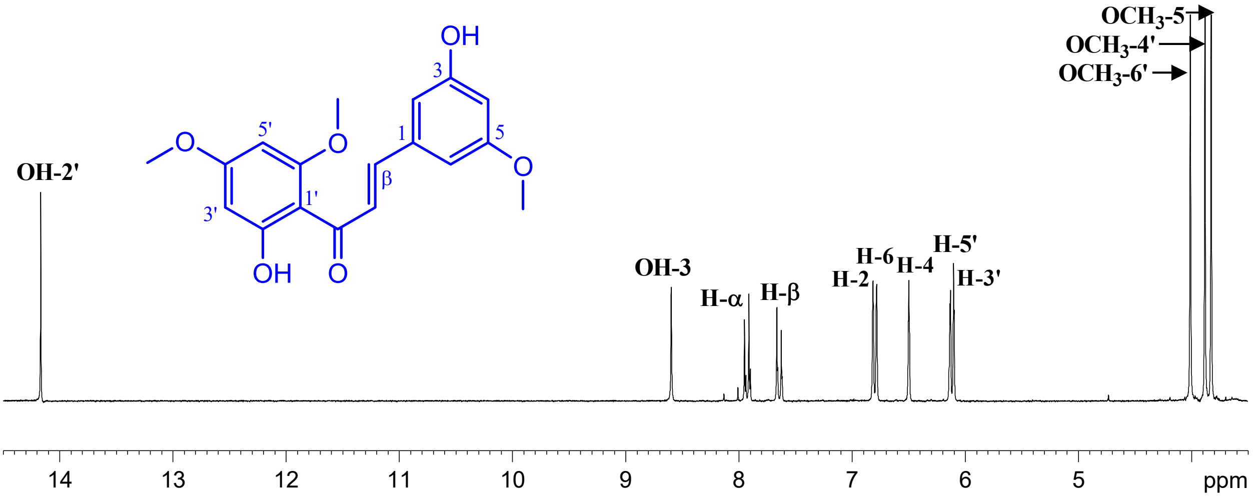

Figure S16. ^{1}H NMR spectrum [400 MHz, solvent $(CD_3)_2CO$] of compound **4**.

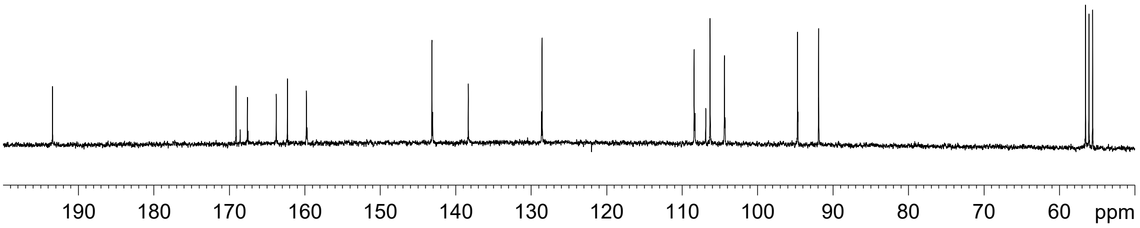

Figure S17. ^{13}C NMR spectrum [100 MHz, solvent $(CD_3)_2CO$] of compound **4**.

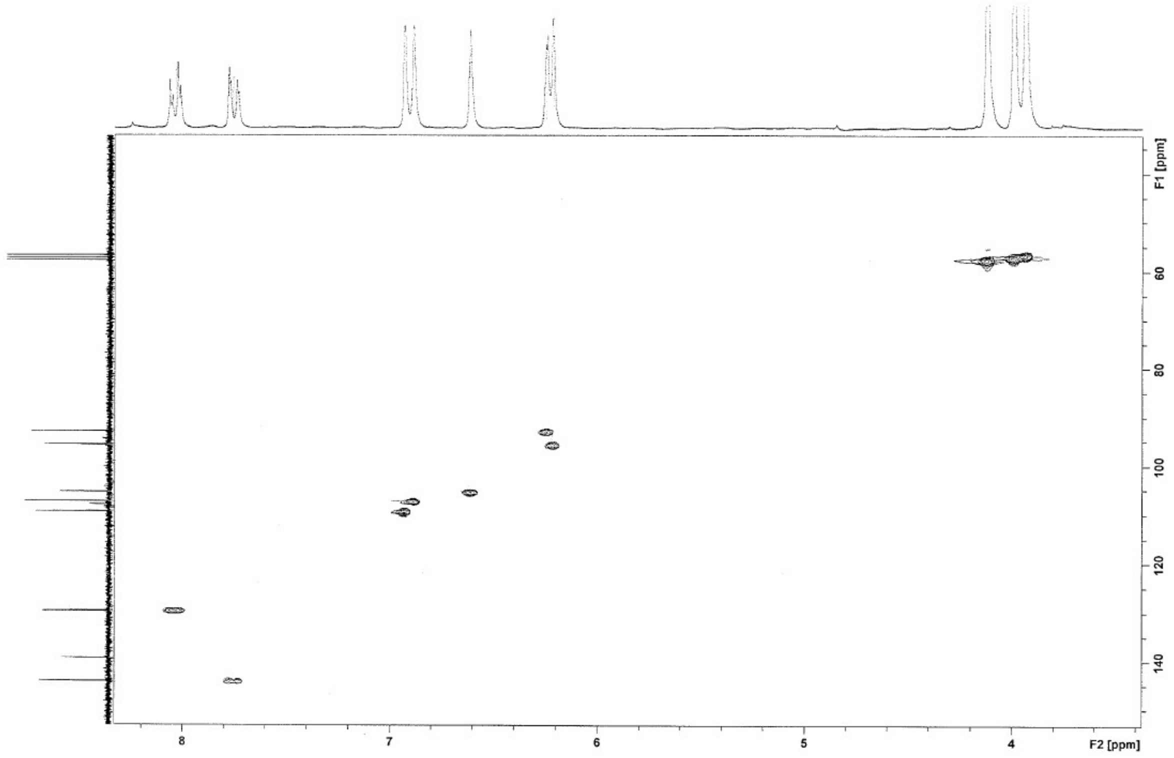

Figure S18. HSQC spectrum [400 MHz, solvent $(CD_3)_2CO$] of compound **4**.

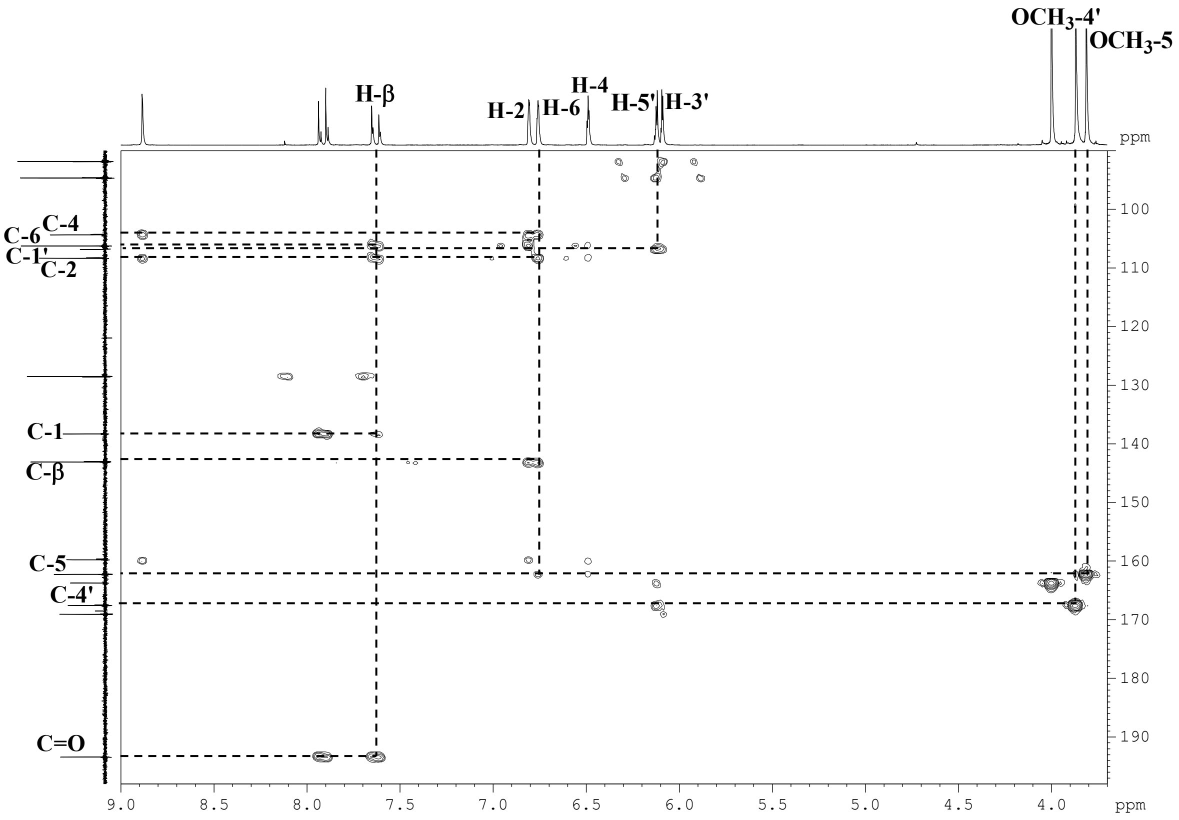

Figure S19. HMBC spectrum [400 MHz, solvent $(CD_3)_2CO$] of compound **4**.

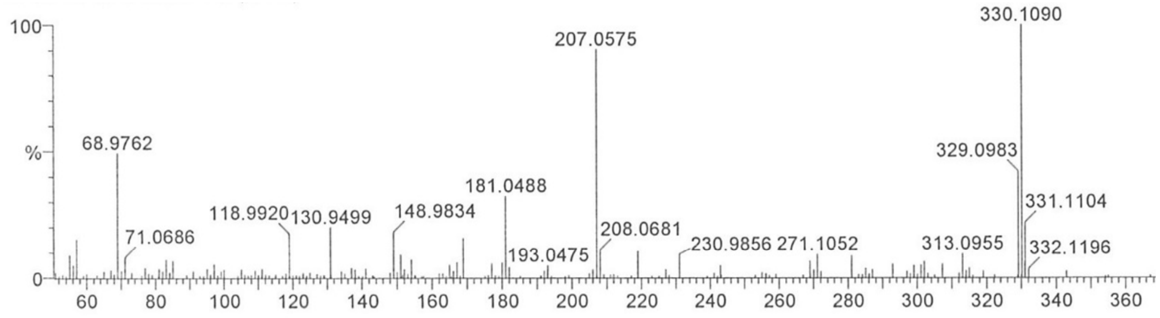

Minimum:	0.10				-1.5		
Maximum:	100.00		5.0	19.0	50.0		
Mass	RA	Calc. Mass	mDa	PPM	DBE	i-FIT	Formula
330.1090	100.00	330.1103	-1.3	-3.9	10.0	2.4	C18 H18 O6

Figure S20. Mass spectrum of compound **4**.

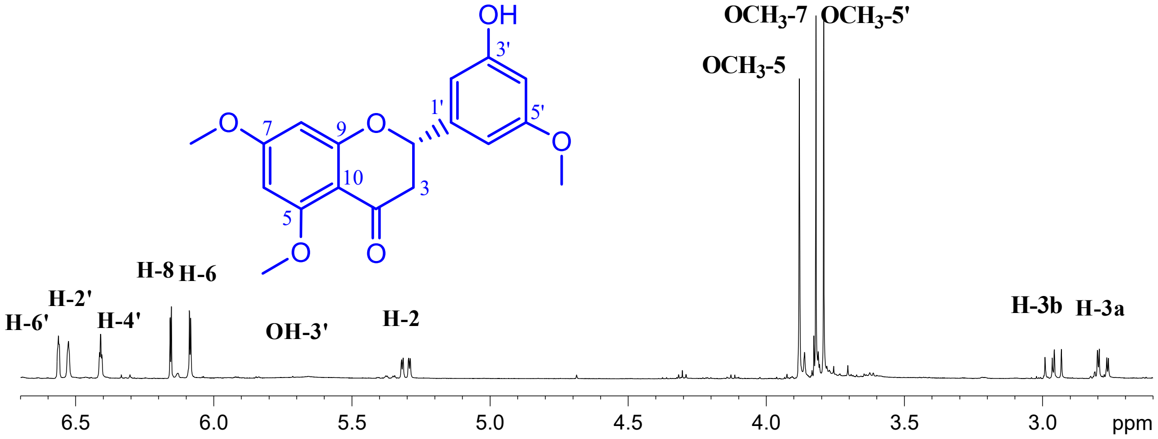

Figure S21. ^{1}H NMR spectrum (400 MHz, solvent $CDCl_3$) of compound **5**.

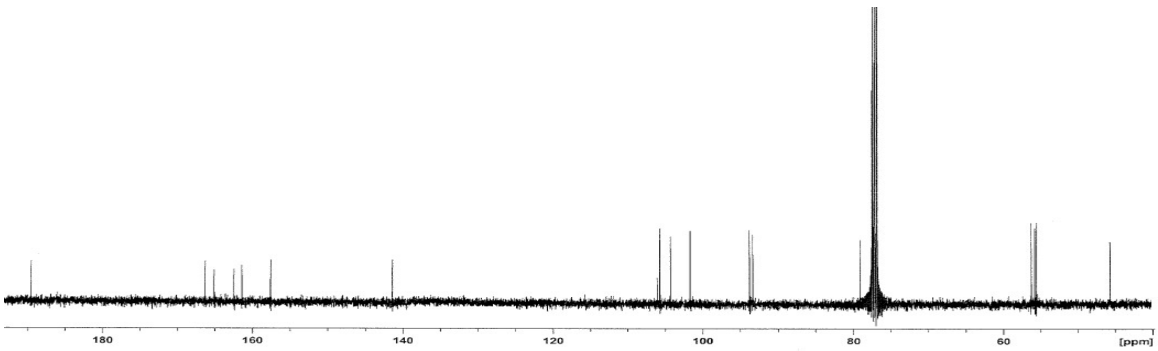

Figure S22. ^{13}C NMR spectrum (100 MHz, solvent $CDCl_3$) of compound **5**.

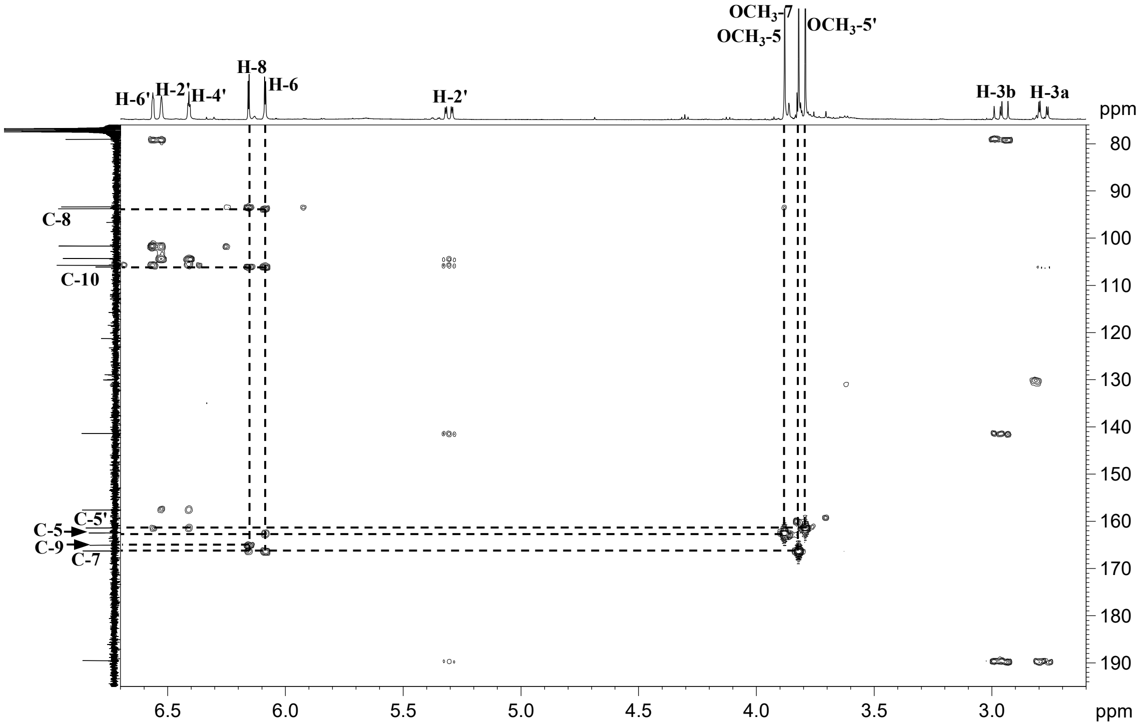

Figure S23. HMBC spectrum (400 MHz, solvent $CDCl_3$) of compound **5**.

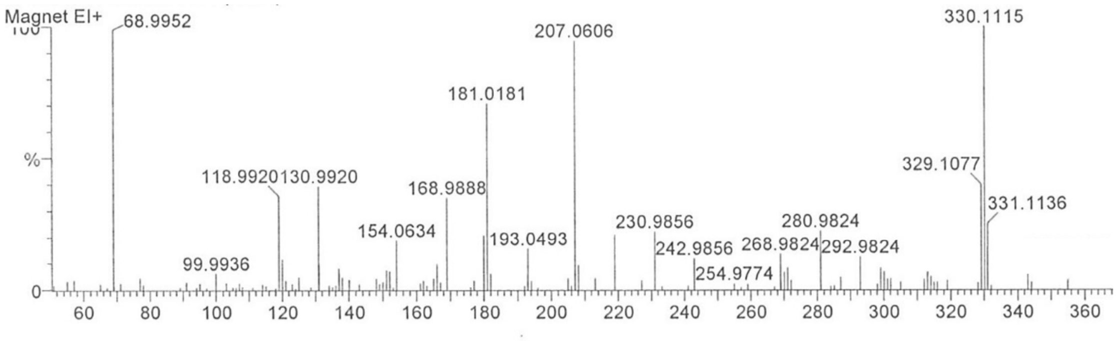

Minimum:	0.10				-1.5		
Maximum:	100.00		5.0	15.0	50.0		
Mass	RA	Calc. Mass	mDa	PPM	DBE	i-FIT	Formula
330.1115	100.00	330.1103	1.2	3.6	10.0	71.2	C18 H18 O6

Figure S24. Mass spectrum of compound **5**.

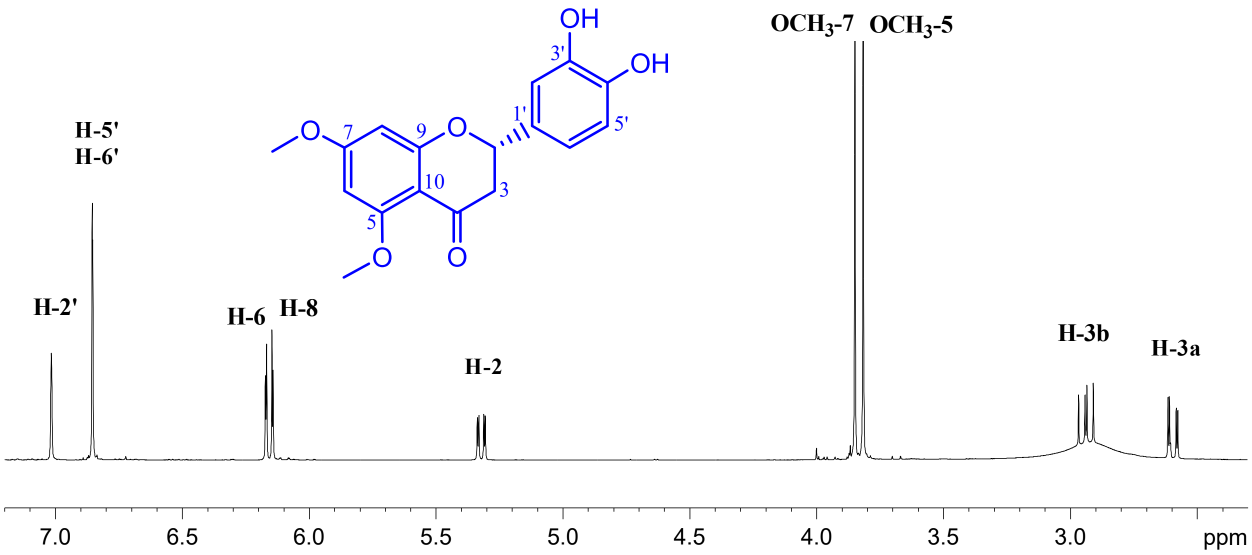

Figure S25. ^{1}H NMR spectrum [400 MHz, solvent $(CD_3)_2CO$] of compound **6**.

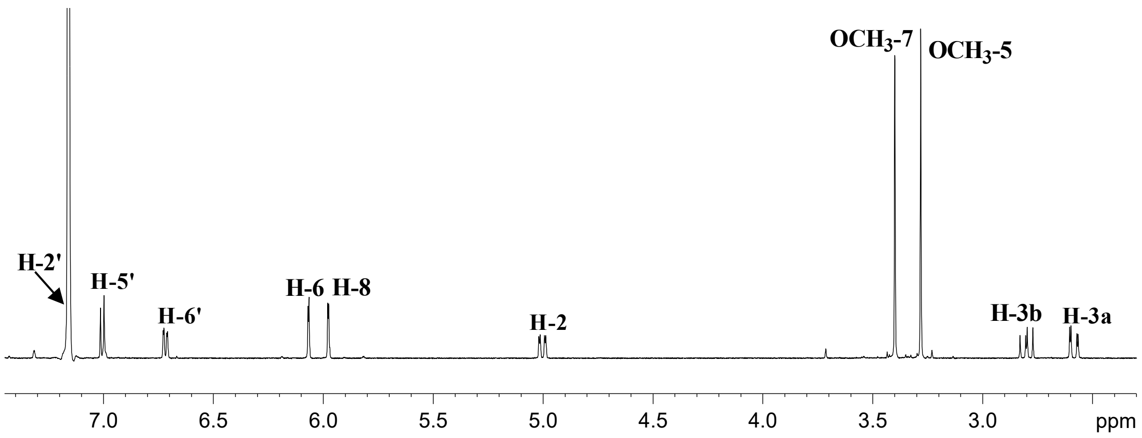

Figure S26. ^{1}H NMR spectrum (500 MHz, solvent C_6D_6) of compound **6**.

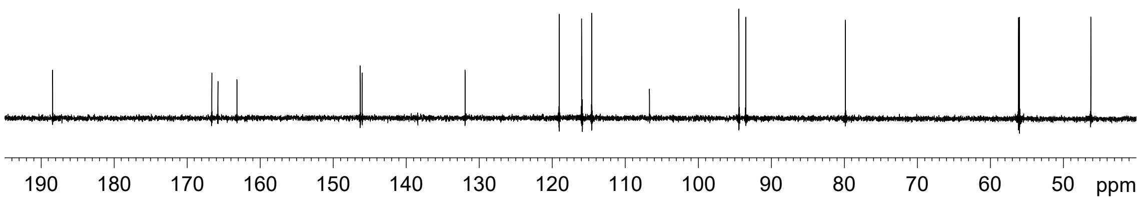

Figure S27. ^{13}C NMR spectrum [100 MHz, solvent $(CD_3)_2CO$] of compound **6**.

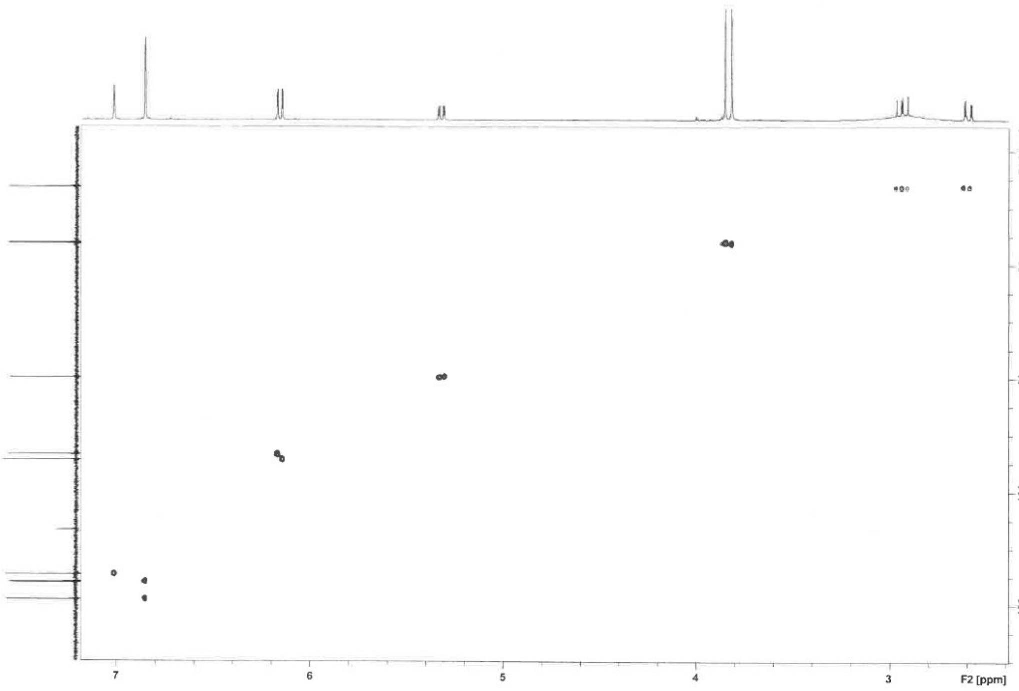

Figure S28. HSQC spectrum [400 MHz, solvent $(CD_3)_2CO$] of compound **6**.

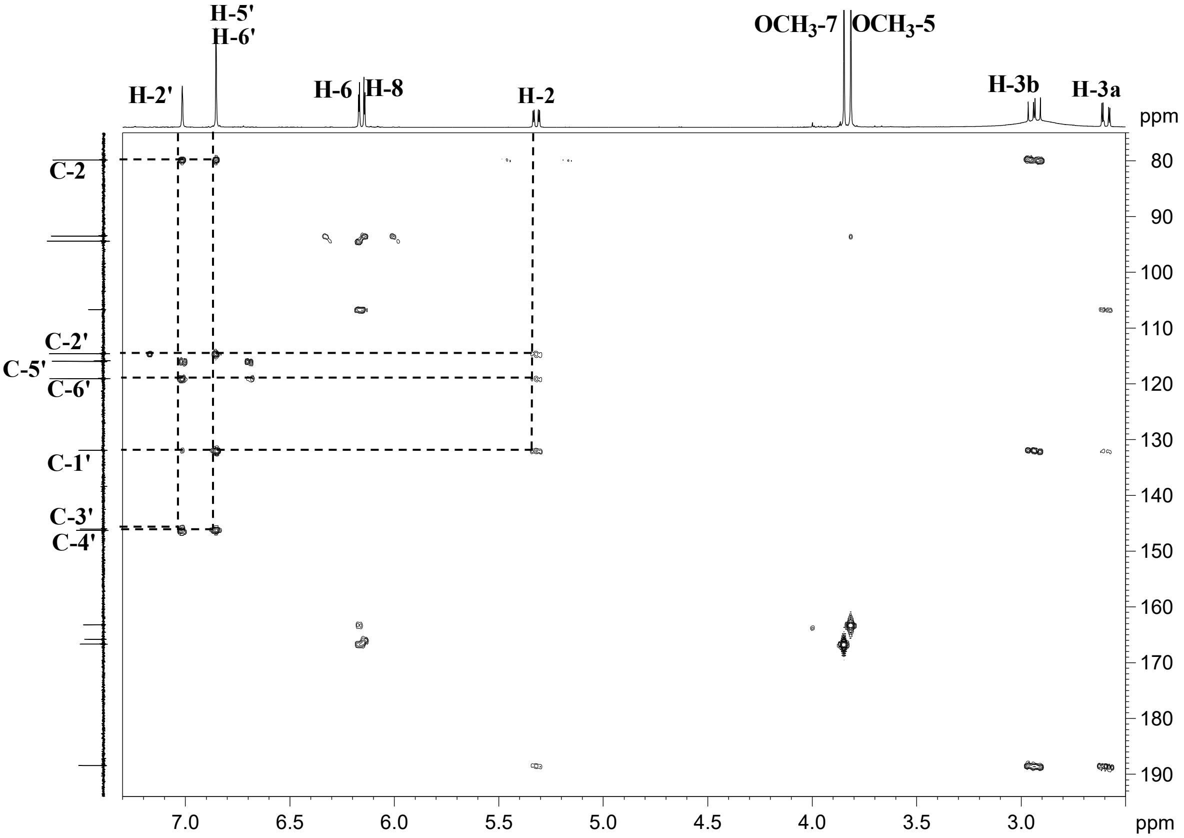

Figure S29. HMBC spectrum [500 MHz, solvent $(CD_3)_2CO$] of compound **6**.

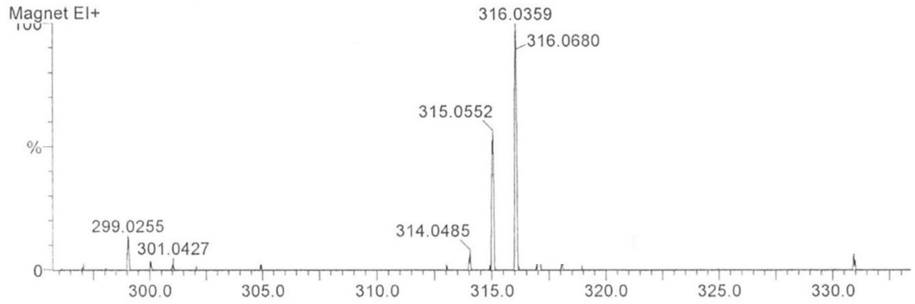

Minimum:	1.00				-1.5	
Maximum:	100.00		5.0	50.0	50.0	
Mass	RA	Calc. Mass	mDa	PPM	DBE	Formula
316.1088	23.61	316.0947	14.1	44.6	10.0	C17 H16 O6

Figure S30. Mass spectrum of compound **6**.

Table S1. *In silico* ADME profile prediction of isolated flavonoids **1-29**.

Table S1. Computational pharmacokinetic parameters (ADME) of isolated flavonoids (**1-29**) from *Piper delineatum*, *P. glabratum* and *P. divaricatum*.

compd	#stars	QPlogBB	QPPCaco	QPPMDCK	QPlogKhsa	QPlogPo/w	QPlogKp	QPlogS	#metab	%HOA	PSA	SASA	mol MW	#rotor	donorHB	accptHB	volume
**1**	0	-1,744	161,65	69,011	-0,022	2,265	-3,299	-3,555	4	79,738	95,0	555,1	286,284	8	2	4	929,3
**2**	0	-1,603	237,596	104,642	-0,008	2,469	-3,031	-3,539	5	83,926	102,8	575,8	316,31	9	2	4,75	995,6
**3**	0	-1,85	162,19	69,259	0,003	2,375	-3,396	-3,815	5	80,409	103,3	592,0	316,31	9	2	4,75	1004,1
**4**	0	-1,325	533,788	250,997	0,179	3,204	-2,441	-4,242	5	94,518	89,0	616,0	330,337	9	1	4,75	1055,4
**5**	0	-0,658	1101,629	549,269	0,131	2,833	-2,392	-4,34	7	100	80,3	596,0	330,337	4	1	5,75	1029,0
**6**	0	-1,091	395,79	181,659	-0,009	2,092	-3,187	-3,984	6	85,687	93,5	570,3	316,31	4	2	5,75	975,5
**7**	0	-1,246	523,193	245,616	0,172	3,118	-2,354	-4,115	4	93,864	80,8	580,9	300,31	8	1	4	981,8
**8**	0	-1,15	523,287	245,664	0,158	3,017	-2,255	-3,869	3	93,271	72,5	543,4	270,284	7	1	3,25	906,5
**9**	0	-0,717	1724,836	891,757	0,206	3,836	-1,398	-4,535	4	100	66,5	604,5	314,337	8	0	4	1032,8
**10**	0	-1,072	779,289	377,823	0,164	3.28	-2,06	-3,865	5	100	88,4	591,3	330,337	9	1	4,75	1045,7
**11**	0	-1,849	201,478	87,56	0,025	2,57	-3,266	-4,044	6	83,236	108,8	627,9	346,336	10	2	5,5	1079,9
**12**	0	-1,129	661,863	316,681	0,175	3,212	-2,111	-4,072	4	96,241	78,0	578,5	300,31	8	1	4	982,3
**13**	0	-2,279	58,093	22,831	-0,2	1,565	-4,193	-3,239	5	67,683	116,5	566,0	302,283	9	3	4,75	950,5
**14**	0	-1,731	161,09	68,752	-0,013	2,324	-3,367	-3,401	5	80,055	102,4	567,6	316,31	9	2	4,75	991,4
**15**	0	-1,768	191,236	82,758	-0,002	2,421	-3,238	-3,779	5	81,955	102,3	589,9	316,31	9	2	4,75	1001,8
**16**	0	-0,86	586,349	277,814	0,035	2,729	-2,546	-2,525	5	92,468	74,0	467,3	272,3	7	1	3,25	849,7
**17**	0	-1,16	335,992	152,184	-0,008	2,034	-3,351	-3,977	7	84,072	94,5	569,8	316,31	4	2	5,75	975,7
**18**	0	-0,836	430,01	198,694	0,136	2,382	-2,931	-3,7	5	88,027	77,8	487,9	256,257	2	1	3,25	815,7
**19**	0	-0,482	1105,422	551,314	0,116	2,653	-2,191	-4,127	5	96,954	63,8	521,6	270,284	2	1	4,25	878,8
**20**	0	-1,162	340,277	154,282	0,002	2,07	-3,326	-4,032	6	84,379	93,6	573,2	316,31	4	2	5,75	980,4
**21**	0	-0,608	1235,294	621,646	0,122	2,856	-2,268	-4,332	7	100	77,5	595,1	330,337	4	1	5,75	1025,7
**22**	0	-0,526	1220,011	613,337	0,125	2,789	-2,179	-4,272	6	100	70,0	558,8	300,31	3	1	5	953,8
**23**	0	-0,935	429,445	198,411	0,152	2,484	-3,032	-3,946	5	88,615	86,1	525,1	286,284	3	1	4	890,8
**24**	0	-0,06	3629,382	1992,796	0,074	3,354	-1,334	-4,166	6	100	57,7	583,4	314,337	3	0	5	1006,2
**25**	0	-0,866	386,611	177,11	0,131	2,367	-2,905	-3,6	2	87,111	77,1	477,7	254,242	2	1	3	796,6
**26**	0	-0,966	385,578	176,598	0,148	2,468	-3,009	-3,845	3	87,685	85,3	514,7	284,268	3	1	3,75	871,4
**27**	0	-1,467	139,157	58,692	-0,009	1,79	-3,899	-3,577	4	75,794	106,8	525,6	300,267	4	2	4,5	892,6
**28**	0	-1,229	191,312	82,794	-0,041	1,787	-3,478	-3,307	3	78,249	96,4	488,6	270,241	3	2	3,75	816,6
**29**	0	-1,427	142,028	60,002	-0,015	1,79	-3,835	-3,468	4	75,949	106,9	519,1	300,267	4	2	4,5	888,1
Range[a]	0 to 5	-3.0 to 1.2	< 25 poor >500 great	< 25 poor >500 great	-1.5 to 1.5	-2 to 6.5	-8.0 to -1.0	-6.5 to 0.5	1.0 to 8.0	< 25 % poor >80% great	7.0 to 200.0	30.0 to 1,000	130.0 to 725.0	0 to 15.0	0 to 6.0	0 to 20.0	500 to 2000

#star (number of property values that fall outside the 95% range of similar values for known drugs), QPlogBB (predicted brain/blood partition coefficient), QPPCaco2 (predicted human epithelial colorectal adenocarcinoma cell lines permeability in nm/s), QPPMDCK (predicted Madin-Darby Canine Kidney permeability in nm/s), QPlogKhsa (prediction of binding to human serum albumin), QPlogPo/w (predicted octanol/water partition coefficient), QPlogKp (skin permeability), QPlogS (predicted aqueous solubility), #metab (number of likely metabolic reactions), %HOA (Percent Human Oral Absorption), PSA (Van der Waals surface area polar nitrogen and oxygen atoms and carbonyl atoms), SASA (total solvent accessible surface area), MW (molecular weight), number of non-trivial, non-hindered ratable bonds). [a] Recommended values.

Experimental part S1. Phytochemical studies

4.5. Extraction and Isolation

The air-dried and powdered leaves of *P. delineatum* (252.2 g) were extracted in a Soxhlet apparatus with 4 L of 96% ethanol (EtOH) until exhaustion. The solvent was evaporated to afford 57.3 g of extract, which was assayed on *G. intestinalis* trophozoites giving a potent activity. Therefore, the ethanolic extract was suspended in water and solvent-solvent partitioned sequentially with dichloromethane (DCM) and ethyl acetate (EtOAc). The organic phases were concentrated under reduced pressure to give DCM (30.0 g) and EtOAc (3.3 g) fractions, whereas the aqueous residue (H_2O) was lyophilized providing the H_2O fraction (20.5 g). Biological evaluation revealed that the organic fractions were active against the strain of *Giardia intestinalis*, and were further investigated

The DCM (30 g) residue was chromatographed on a silica gel column, using mixtures of hexanes/EtOAc of increasing polarity (10:0 to 0:10) as eluent to afford nineteen sub-fractions, which were combined based on their TLC profile in sub-fractions D1-D10. Giardicidal activity revealed that sub-fractions D3-D8 were active against the strain of *G. intestinalis*, and were subjected to several chromatography steps until obtaining the pure compounds. Sub-fraction D3 (0.5 g) was chromatographed on Sephadex LH-20 (hexanes/$CHCl_3$/MeOH, 2:1:1), affording sub-fractions D3A-D3E. Sub-fraction D3C (76.9 mg) was chromatographed by CPTLC, using mixtures of hexanes/EtOAc of increasing polarity (6:4 to 4:6) to afford sub-fractions D3C1- D3C6. Sub-fraction D3C5 (16.4 mg) was further purified by preparative TLC (hexanes/EtOAc, 6:4) to give compounds **9** (5.2 mg) and **7** (1.2 mg). Sub-fraction D3D (61.5 mg) was chromatographed by CPTLC, using mixtures of hexanes/EtOAc of increasing polarity (6:4 to 4:6) to afford sub-fractions D3D1-D3D5. Sub-fraction D3D3 (10.8 mg) was

further purified by preparative TLC (CH_2Cl_2/acetone 9:1) to give compound **8** (9.3 mg). Sub-fraction D4 (1.8 g) was chromatographed on Sephadex LH-20 (hexanes/$CHCl_3$/MeOH, 2:1:1), affording sub-fractions D4A-D4F. Sub-fraction D4E (196.1 mg) was chromatographed by CPTLC (hexanes/isopropanol of increasing polarity, 10:0 to 9:1) to afford sub-fractions D4E1-D4E3. Sub-fraction D4E1 (17.1 mg) was further purified by preparative TLC (hexanes/isopropanol 9:1) to give compounds **4** (1.2 mg) and **10** (5.9 mg). Sub-fraction D4F (196.4 mg) was chromatographed by CPTLC (CH_2Cl_2-acetone, 10:0 to 1:1) to give sub-fractions D4F1- D4F7. Sub-fraction D4F7 (9.1 mg) was further purified by preparative TLC (hexanes/isopropanol, 9:1) to yield compounds **12** (4.3 mg) and **16** (2.9 mg). Sub-fraction D5 (1.66 g) was chromatographed on Sephadex LH-20 ($CHCl_3$/MeOH, 1:1), affording sub-fractions D5A-D5F. Sub-fraction D5E (360.5 mg) was chromatographed by CPTLC (CH_2Cl_2/acetone of increasing polarity, 10:0 to 1:1) to afford sub-fractions D5E1-D5E9. Sub-fraction D5E8 (24.1 mg) was further purified by preparative TLC (hexanes/isopropanol, 9:1) to yield compound **7** (12.7 mg). Sub-fraction D5F (9.8 mg) was chromatographed by preparative TLC (CH_2Cl_2/isopropanol, 9:1) to yield compounds **4** (4.6 mg) and **12** (2.2 mg). Sub-fraction D6 (1.61 g) was chromatographed on Sephadex LH-20 ($CHCl_3$/MeOH, 1:1), affording sub-fractions D6A-D6G. Sub-fraction D6D (89.9 mg) was chromatographed by CPTLC (CH_2Cl_2/acetone of increasing polarity, 10:0 to 8:2) to afford sub-fractions D6D1-D6D5. Sub-fraction D6D3 (20.6 mg) was further purified by preparative TLC (CH_2Cl_2/1,4-dioxane, 9:1) to yield compounds **1** (5.7 mg) and **3** (3.7 mg). Sub-fraction D6E (202.0 mg) was chromatographed by CPTLC (CH_2Cl_2/acetone of increasing polarity, 10:0 to 8:2) to afford sub-fractions D6E1-D6E6. Sub-fraction D6E4 (8.8 mg) was further purified by preparative TLC (CH_2Cl_2/1,4-dioxane, 9:1) to yield compounds **2** (1.9 mg) and **11** (2.4 mg). Sub-fraction D6F (615.4 mg) was chromatographed by CPTLC (CH_2Cl_2/acetone

of increasing polarity, 10:0 to 8:2) to afford sub-fractions D6F1-D6F8. Sub-fraction D6F2 (25.9 mg) was identified as compounds **7**, whereas sub-fractions D6F4 (27.6 mg) and D6F5 (18.9) were further purified by preparative TLC (CH_2Cl_2/1,4-dioxane, 8:2) to yield compounds **4** (19.6 mg) and **12** (9.4 mg), respectively. Sub-fraction D7 (1.98 g) was chromatographed on Sephadex LH-20 ($CHCl_3$/MeOH, 1:1), affording sub-fractions D7A-D7I. Sub-fraction D7F (532.0 mg) was chromatographed by CPTLC (hexanes/isopropanol of increasing polarity, 10:0 to 8:2) to afford sub-fractions D7F1-D7F3. Sub-fractions D7F1 (229.8 mg) and D7F3 (65.8 mg) were identified as compounds **1** and **3**, respectively. Sub-fraction D7F (27.0 mg) was further purified by preparative TLC (2 x development, hexanes/isopropanol, 8:2) to yield compounds **11** (8.0 mg) and **14** (16.8 mg). Sub-fraction D8H (5.44 g) was chromatographed on Sephadex LH-20 ($CHCl_3$/MeOH, 1:1), affording sub-fractions D8A-D8I. Sub-fraction D8B (34.2 mg) was chromatographed by preparative TLC (CH_2Cl_2/acetone 1:1) to yield compounds **3** (5.5 mg), **10** (9.3 mg) and **12** (2.7 mg). Sub-fraction D8F (506.0 mg) was chromatographed by CPTLC (CH_2Cl_2-acetone, 10:0 to 7:3), giving sub-fractions D8F1-D8F9. Sub-fraction D8F2 (7.5 mg) was further purified by preparative TLC (hexanes/isopropanol, 4:1) to yield compounds **13** (3.1 mg) and **14** (1.8 mg). Sub-fraction D8F7 (52.9 mg) was identified as compound **3**. Sub-fraction D8F8 (20.5 mg) was further purified by preparative TLC (CH_2Cl_2/acetone 1:1) to give compound **11** (15.3 mg). Sub-fraction D8F9 (17.4 mg) was further purified by preparative TLC (CH_2Cl_2/acetone 1:1) to give compound **5** (7.5 mg). Sub-fraction D8H (30.9 mg) was chromatographed by preparative TLC (CH_2Cl_2/acetone 1:1) to yield compounds **19** (21.5 mg) and **24** (3.4 mg).

Based these anti-parasitic results in which the activity seems to be related to the presence of flavonoids, and in order to complete the phytochemical study, the inactive sub-fractions (D1, D2, D9 and D10) were analyzed by ^{1}H NMR and TLC, revealing the

presence of flavonoids in sub-fractions D9 and D10, which were further investigated. Sub-fraction D9 (3.7 g) was chromatographed on Sephadex LH-20 ($CHCl_3$/MeOH, 1:1), affording sub-fractions D9A-D9H. Sub-fraction D9G (15.4 mg) was further purified by preparative TLC (CH_2Cl_2/isopropanol, 9:1) to afford compound **17** (7.2 mg). Sub-fraction D10 (3.9 g) was chromatographed on Sephadex LH-20 ($CHCl_3$/MeOH, 1:1) affording sub-fractions D10A-D10E. Fraction D10B (69.8 mg) was chromatographed by CPTLC (CH_2Cl_2/acetone, 10:0 to 7:3) affording sub-fractions D10B1-D10B5. Sub-fraction D10B3 (16.4 mg) was further purified by preparative TLC (CH_2Cl_2/acetone, 9:1) to afford compounds **20** (6.1 mg) and **17** (1.2 mg). Sub-fraction D10E (492.0 mg) was chromatographed by CPTLC (CH_2Cl_2/acetone, 10:0 to 7:3) to afford sub-fractions D10E1-D10E12. Sub-fraction D10E6 (16.4 mg) was further purified by preparative TLC (CH_2Cl_2/acetone, 9:1) to afford compounds **20** (6.4 mg) and **21** (3.4 mg), whereas sub-fraction D10E8 (21.2 mg) affords compounds **21** (2.4 mg) and **22** (8.3 mg) by preparative TLC (hexanes/isopropanol, 8:2).

The EtOAc fraction (3.3 g) was chromatographed on a silica gel column, using mixtures of CH_2Cl_2/EtOAc of increasing polarity (200 mL of 8:2, 6:4, 4:6, 2:8 and 0:10) and MeOH as eluent to afford sub-fractions (E1-E6). Preliminary giardicidal activity analysis revealed that E2 and E3 sub-fractions were active against the strain of *G. intestinalis*, which were further investigated. Sub-fraction E2 (15.6 mg) was further purified by preparative TLC (hexanes/isopropanol, 8:2) to give compounds **1** (2.1 mg), **7** (1.6 mg) and **3** (5.3 mg). Sub-fraction E3 (314 mg) was chromatographed on silica gel by CPTLC, using an isocratic mixture (CH_2Cl_2/EtOAc, 7:3) as eluent to give sub-fractions E3A-E3C (C1-C3). Sub-fraction E3A (8.7 mg) was further purified on silica gel by preparative TLC (2 x development, CH_2Cl_2/EtOAc, 7:3) to give compounds **1** (2.1 mg), **7** (1.6 mg) and **3** (5.3 mg). Sub-fraction E3B (173 mg) was chromatographed on silica gel by CPTLC, using an isocratic mixture ($CHCl_3$/isopropanol, 19:1) as eluent

to give sub-fractions E3B1-E3B5. Sub-fraction E3B3 (17.4 mg) was further purified on silica gel by preparative TLC (3 x development, $CHCl_3$/isopropanol, 9:1) to give compound **17** (7.5 mg).

The air-dried fruits of *P. glabratum* (15.9 g) were ground and extracted with EtOH 70% (2 x 400 mL) by maceration. Evaporation of the solvent under reduced pressure provided 3.2 g of crude extract, which was successively partitioned into CH_2Cl_2/H_2O (1:1, v/v) solution. Removal of the CH_2Cl_2 from the organic-soluble extract under reduced pressure yielded 2.1 g of residue, whereas the aqueous residue (H_2O) was lyophilized providing the H_2O fraction (0.5 g). The CH_2Cl_2 residue was chromatographed on a silica gel column, using mixtures of hexanes/EtOAc of increasing polarity (200 mL of 9:1, 8:2, 7:3, 6:4, 5:5, 4:6, 3:7, 2:8 and 0:10) as eluent to afford nine fractions (A-I). Preliminary ^1H NMR and TLC analysis revealed the presence of flavonoids in some of them, which were further investigated. Fraction C (209.9 mg) was chromatographed on Sephadex LH-20 ($CHCl_3$/MeOH, 1:1) to afford nine sub-fractions C1-C9. Sub-fractions C4 (11.7 mg) and C6 (8.0 mg) were further purified by HPTLC (hexanes/isopropanol, 9:1) to give compound **18** (7.4 mg) and compounds **28** (3.6 mg), respectively. Fraction E (172.2 mg) was chromatographed on Sephadex LH-20 ($CHCl_3$/MeOH, 1:1) to afford four sub-fractions E1-E4. Subfraction E6F (22.9 mg) was further purified by HPTLC (hexanes/EtOAc, 4:6) to give compounds **25** (3.3 mg).

The air-dried and powdered leaves of *P. divaricatum* (343.2 g) were extracted in a Soxhlet apparatus with 96% ethanol (4 x 4 L) until exhaustion. The solvent was evaporated to afford 75.7 g of crude extract. The ethanolic extract (EtOH) was suspended in water and solvent-solvent partitioned sequentially with dichlorometane (DCM) and ethyl acetate (EtOAc). The resulting organic phases were concentrated under reduced pressure to give DCM (29.8 g) and EtOAc (0.6 g) fractions, whereas the

aqueous residue (H_2O) was lyophilized providing the H_2O fraction (12.1 g). The CH_2Cl_2 fraction was chromatographed on a silica gel column, using mixtures of hexanes/EtOAc of increasing polarity (2 L, 10:0 to 0:10) as eluent to afford fractions (A-G). Preliminary ^1H NMR and TLC analysis revealed the presence of flavonoids in fractions D (hexanes/EtOAc, 7:3), F (hexanes/EtOAc, 3:7) and G (hexanes/EtOAc, 0:10), and were further investigated. Fraction D (4.8 g) was chromatographed on Sephadex LH-20 ($CHCl_3$/MeOH, 1:1) to afford fractions D1-D3 based on TLC analysis. Sub-fraction D3 (1.1 g) was chromatographed by a silica gel column, using mixtures of hexanes/EtOAc of increasing polarity (10:0 to 5:5) as eluent to afford fractions (D3A-D3I). An aliquot of sub-fractions D3G (11.5 mg), D3H (11.2 mg) and D3I (9.8 mg) were further purified by HPTLC (hexanes/isopropanol, 9:1) to give compounds **26** (3.5 mg), **29** (4.5 mg) and **23** (7.8 mg), respectively. Fraction F (1.5 g) was chromatographed on Sephadex LH-20 ($CHCl_3$/MeOH, 1:1) to afford sub-fractions F1-F7. Sub-fraction F4 (97.7 mg) was chromatographed by CPTLC, using mixtures of hexanes/isopropanol of increasing polarity (9.5:0.5 to 8.5:1.5) as eluent to afford sub-fractions F4A-F4F. Sub-fraction F4D (7.6 mg) was further purified by HPTLC ($CHCl_3$/EtOH, 9.5:0.5) to give compound **27** (4.7 mg), and sub-fraction F-7 (9.7 mg) yielded compound **6** (4.4 mg) by HPTLC ($CHCl_3$/EtOH, 9.5:0.5). Fraction G (288.3 mg) was chromatographed on Sephadex LH-20 ($CHCl_3$/MeOH, 1:1) to afford sub-fractions G1-G4. Sub-fractions G3 (31.9 mg) and G4 (14.1 mg) were purified by HPTLC (CH_2Cl_2/acetone 8:2) to give compounds **6** (8.9 mg) and **15** (4.4 mg).
